# Low incidence of microsatellite instability in gastric cancers and its association with the clinicopathological characteristics: a comparative study

**DOI:** 10.1038/s41598-023-48157-7

**Published:** 2023-12-08

**Authors:** Fateme Fooladi Talari, Ali Bozorg, Sirous Zeinali, Mohammadreza Zali, Zhale Mohsenifar, Hamid Asadzadeh Aghdaei, Kaveh Baghaei

**Affiliations:** 1 Biotechnology Department, College of Science, University of Tehran, Tehran, Iran; 2 Dr. Zeinali’s Medical Genetics Laboratory, Kawsar Human Genetics Research Center, Tehran, Iran; 3 Department of Molecular Medicine, Biotechnology Research Center, Pasteur Institute of Iran, Tehran, Iran; 4 Research Institute for Gastroenterology and Liver Diseases, Gastroenterology and Liver Diseases Research Center, Shahid Beheshti University of Medical Sciences, Tehran, Iran; 5 Department of Pathology, School of Medicine, Taleghani Hospital, Shahid Beheshti University of Medical Sciences, Tehran, Iran

**Keywords:** Gastric cancer, Cancer genomics, Cancer epidemiology

## Abstract

Gastric cancer is a complex heterogeneous disease with different molecular subtypes that have clinical implications. It is characterized by high mortality rates and limited effective therapies. Microsatellite instability (MSI) has been recognized as a subgroup with a good prognosis based on TCGA and ACRG categorizations. Besides its prognostic and predictive value, gastric cancers with high MSI exhibit different clinical behaviors. The prevalence of high MSI has been assessed in gastric cancer worldwide, especially in East Asia, but there is a lack of such information in the Middle East. Therefore, this study aimed to investigate the incidence and status of MSI in Iranian gastric cancer patients using 53 samples collected from 2015 to 2020 at Taleghani Hospital Medical Center. DNA from tumoral and normal tissues were extracted and assessed through multiplex-PCR based on five mononucleotide repeats panel. Clinicopathological variables, including age, sex, Lauren classification, lymph node involvement, TNM stage, differentiation, localization, and tumor size, were also analyzed. With 2 males and 2 females, high microsatellite instability represented a small subgroup of almost 7.5% of the samples with a median age of 60.5 years. High microsatellite instability phenotypes were significantly associated with patients aged 68 years and older (p‑value of 0.0015) and lower lymph node involvement (p‑value of 0.0004). Microsatellite instability was also more frequent in females, with distal gastric location, bigger tumor size, and in the intestinal type of gastric cancer rather than the diffuse type.

## Introduction

Gastric cancer (GC) is the second most common cancer type and the leading cause of cancer-related death in Iran, with approximately 14,656 new cases (9599 males, 5057 females) reported in 2020, as per Globocan 2020 report^1^.

In the early stages, GC develops with mild gastrointestinal symptoms. Therefore, most patients are usually diagnosed in advanced stages when therapeutic approaches fail, leading to poor prognosis and overall survival^2^. Various treatment approaches have been employed globally, but not all patients benefit from adjuvant chemotherapy, and adverse effects have been reported^3^. Furthermore, GC has been identified as a complex and heterogeneous disease. Evidence indicates that GC prognosis and treatment outcomes depend not only on the tumor stage, but also on the molecular and genetic features^4,5^. Different classifications have been introduced for GC. Lauren histological classification, comprised of two intestinal and diffuse subtypes, was first introduced in 1965^6^ and recently updated for more clarification of histological features^7^. Another classification based on morphological patterns, including mucinous (colloid), tubular, papillary, and mixed carcinoma, but with no correlations with prognosis, has been suggested by WHO^8^. However, due to their limited clinical applications, new insightful molecular classification of GC has been provided to better direct clinical decisions. Advances in high-throughput molecular analysis, like next-generation sequencing (NGS), and microarray, have led to new insights for discovering novel predictive markers to improve targeted therapy of GC at the edge of precision medicine^9^.

In 2014 and 2015, The Cancer Genome Atlas (TCGA) and Asian Cancer Research Group (ACRG) respectively presented different molecular classifications of each of the four GC subgroups based on a meta-analysis of the patients. TCGA classified GC into four groups: EBV (Epstein-Barr virus), MSI (microsatellite instability), CIN (chromosomal instability), and GS (genomically stable)^10^. Another four molecular subtypes have been identified by ACRG as MSI, MSS/TP53+ (microsatellite stable with active TP53), MSS/TP53− (microsatellite stable with inactive TP53), and MSS/EMT (microsatellite stable with epithelial-mesenchymal transition (EMT) signature)^11^. This suggests that MSI is the only subtype common to both newly identified molecular classifications.

**Figure 1:**
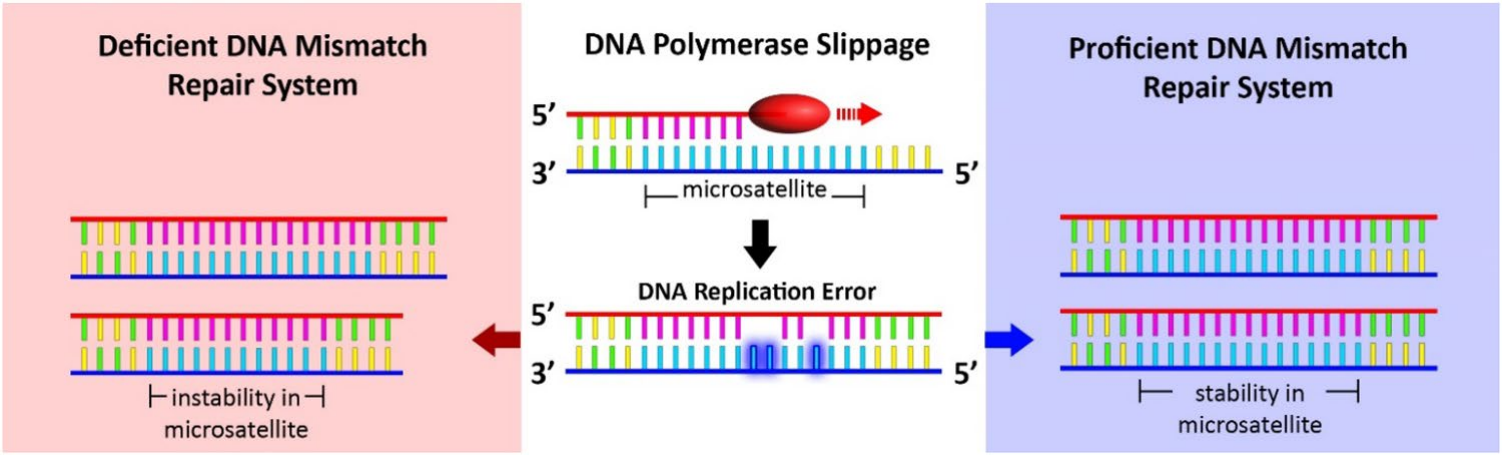
Schematic diagram of MSS and MSI-H/dMMR. In MSI-H status, the error arising from DNA polymerase slippage would remain (original image).

MSI is a genetic instability marker involved in different types of cancer and defines changes in the length of the repeats of microsatellite regions of DNA^12^. Coding and non-coding regions of the genome contain a considerable number of microsatellites that, based on their repetitive nature, would be susceptible to shortening or lengthening due to DNA polymerase slippage during the replication process. The mismatch repair (MMR) system prevents the accumulation of such mismatch errors, but when MMR is deficient, errors accumulate in repetitive microsatellite regions, and eventually, MSI phenotype appears (Fig. 1)^13,14^. It has been shown in recent studies that MSI status could be a marker of immunotherapy. DNA polymerase slippage could lead to the insertion and deletion of nucleotides in repeat regions of DNA sequence. In proficient cells, these errors would be corrected with repair machinery. However, in deficient cells, such errors would not be corrected due to the defective MMR genes, leading to instability in microsatellite regions^15^. Accordingly, novel peptides would be expressed as neoantigens due to the translation of such genes with MSI frameshift mutations, resulting in immune response and PD-1 ligand upregulation. Tumor cells can then escape immune response by triggering T cell death with these ligands. The anti-PD-1 antibodies bind the programmed cell death 1 (PD-1) receptor and prevent the activation of programmed cell death by the PD-1 ligand^16^. The positive influence of pembrolizumab monotherapy compared with toxic chemotherapy in MSI-H GC patients with programmed cell death-ligand 1 (PD-L1) has been reported in KEYNOTE-062 and KEYNOTE-177 studies^17,18^.

In general, clinicopathological features of MSI-H GC have been associated with the female sex, older ages, distal stomach location, and intestinal subtype of the Lauren classification. However, as such assessments have not been conducted evenly across the nations, more investigation would still be required^19^. Herein, the incidence of MSI status and its association with clinical and pathological information has been assessed as a retrospective study in Iranian GC patients.

## Materials and methods

**Figure 2:**
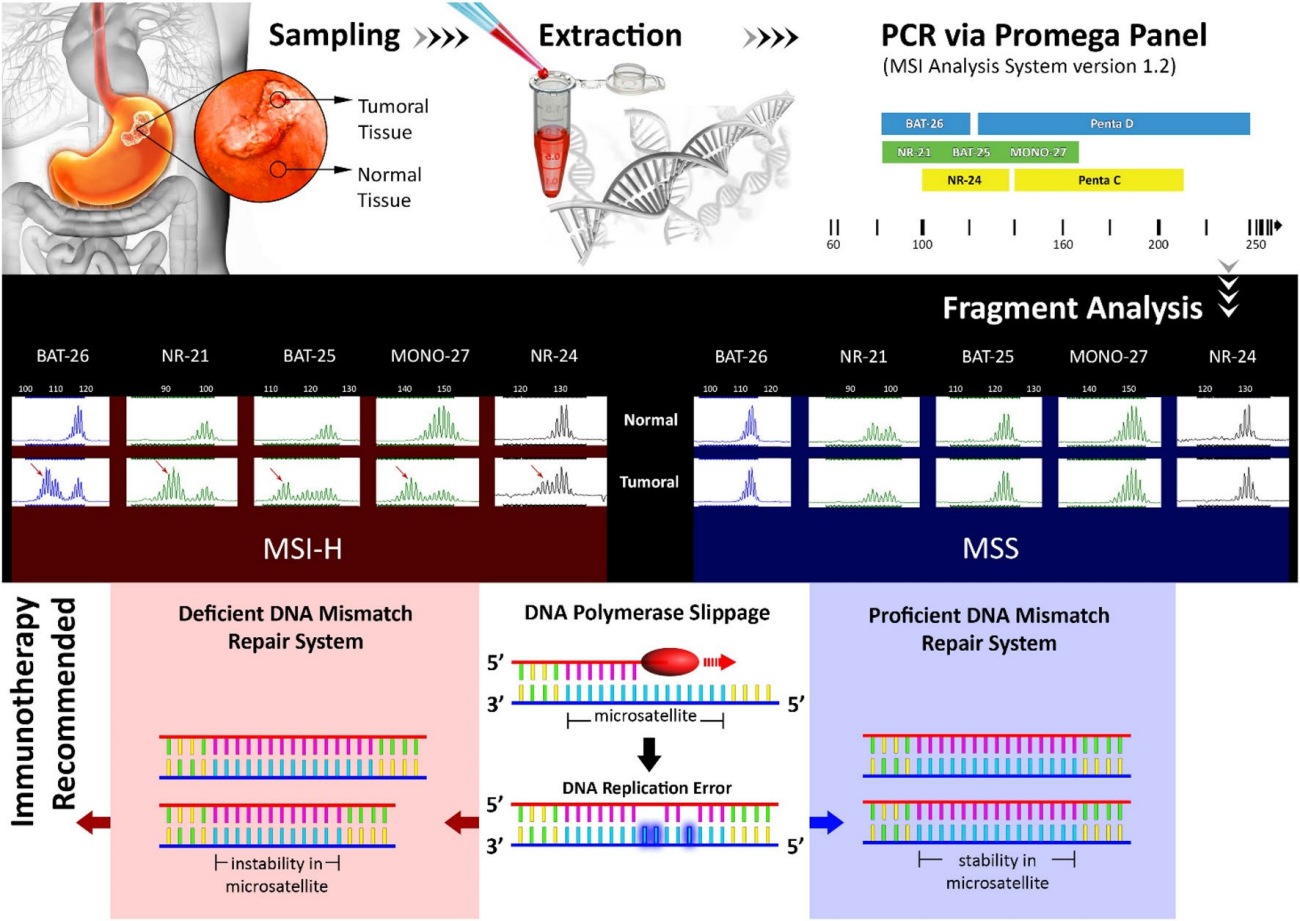
Workflow followed in this study for MSI evaluation based on a multiplex PCR and Fragment Analysis (original image).

All experiments and methods employed in this study were performed in accordance with the relevant guidelines and regulations. The schematic of the workflow followed in this study, including sampling from the patients, DNA extraction, fragment analysis, and statistical analysis for MSI detection, is demonstrated in Fig. 2.

### Patients

This retrospective study comprised 74 GC patients admitted to Taleghani Hospital (Tehran Province, Iran) and underwent surgical resection from 2015 through 2020. The study was approved by the Ethics Committee of Research Institute for Gastroenterology and Liver Diseases, Shahid Beheshti Medical University (No. IR.SBMU.RIGLD.REC.1399.048). Informed consent was obtained from all participants included in this study.

Tumoral and adjacent normal Formalin-fixed paraffin-embedded (FFPE) tissue samples were collected for each patient and cut into eight micro-thick pieces of 5 sections. The criteria for participant inclusion were the existence of both tumoral and normal tissues, pathologically confirmed GC, acceptance of surgical treatment, as well as the accessibility of pathological and clinical data needed for statistical analysis, including sex, age, Lauren classification, TNM stage, lymph node metastasis, degree of differentiation, size of tumor, degree of infiltration, and clinical disease stage. The sample microscope slides were prepared and examined by a pathologist to differentiate the tumoral tissue from the adjacent normal mucosa.

### DNA extraction

Genomic DNA was extracted from the FFPE sections of the patients' tumoral and paired normal tissues using the QIAamp DNA FFPE Tissue Kit (QIAGEN GmbH, Germany). The extracted DNA was eluted with 100 μL of Tris buffer (pH 7.5) and the DNA quantity was determined using Nanodrop 2000 (Thermo Fisher Scientific Inc, USA). The quality of DNA was also assessed by measuring the A260/A280 ratio. Samples with concentrations higher than 5 ng/μL were included.

### MSI detection

All specimens were assessed via the Promega MSI kit containing mononucleotide repeats, namely BAT-25, BAT-26, NR-21, NR-24, MONO-27, and two pentanucleotide repeat markers of Penta C and Penta D as the internal controls. Polymerase chain reaction (PCR) amplification was performed using an ABI 2720 Thermal Cycler (Applied Biosystems, CA, USA) in a 10 μl container containing 5.85 μl Nuclease-Free Water, 1 μl Gold STHR 10X Buffer, 1 μl MSI 10× Primer Pair Mix, 0.15 μl AmpliTaq Gold® DNA polymerase (5 u/μl) with at least 1–2 ng DNA. The PCR products were separated and detected by an ABI 3130 genetic analyzer (Applied Biosystems, CA, USA), and GeneMarker Software version 1.95 (SoftGenetics LLC, USA) was employed to analyze the data. The MSI status was then assessed by considering the shifts in the allelic position of the microsatellite locus in the tumor tissue and its adjacent normal tissue, as well as changes in the electropherograms of microsatellite markers. In such analysis, the samples with no unstable microsatellite loci were considered stable (MSS), those possessed one unstable locus classified as MSI-Low (MSI-L), and the samples revealed size shifts in two or more markers in the tumoral tissue compared to the adjacent normal tissue categorized as MSI-high (MSI-H).

### Statistical analysis

Statistical analysis was performed using SPSS 26.0 (SPSS Inc., Chicago, IL, USA) and GraphPad Prism 8.4.0. Chi-square test (Fisher exact test), t-test, and one-way ANOVA were applied to calculate P values and analyze the relationship between clinicopathologic data and MSI or MSS in GC. Given that MSI-L samples demonstrate similar pathological behavior to MSS samples, MSS and MSI-L samples were combined for sensitivity and specificity evaluations in subsequent analyses.

## Results

### Samples characteristics

Tumoral and adjacent normal FFPE tissues collected from 74 patients were assessed. Following the described exclusion criteria, 21 samples were excluded. In the remaining 53 samples, the mean age was 62, ranging from 23 to 101. The included participants were then categorized based on various factors, including gender, age, Lauren classification, TNM stage, Lymph node status, differentiation, localization, and size. It was concluded that the largest subgroup in each category was respectively male (64%), < 68 years old (66%), intestinal type (68%), stage III pT3(62%), N0 and N3 (30%), poorly differentiated G3 (52%), distal location (57%), and tumors smaller than5 cm (64%).

### MSI status and its association with clinicopathologic characteristics

The MSI phenotype was determined using the Promega MSI Analysis System on 53 gastric cancer patients. The MSI status was identified by comparing the electropherogram of tumor with that of its corresponding normal tissues. Samples exhibiting instability in microsatellite markers of more than 30% were classified as MSI-H.

**Figure 3:**
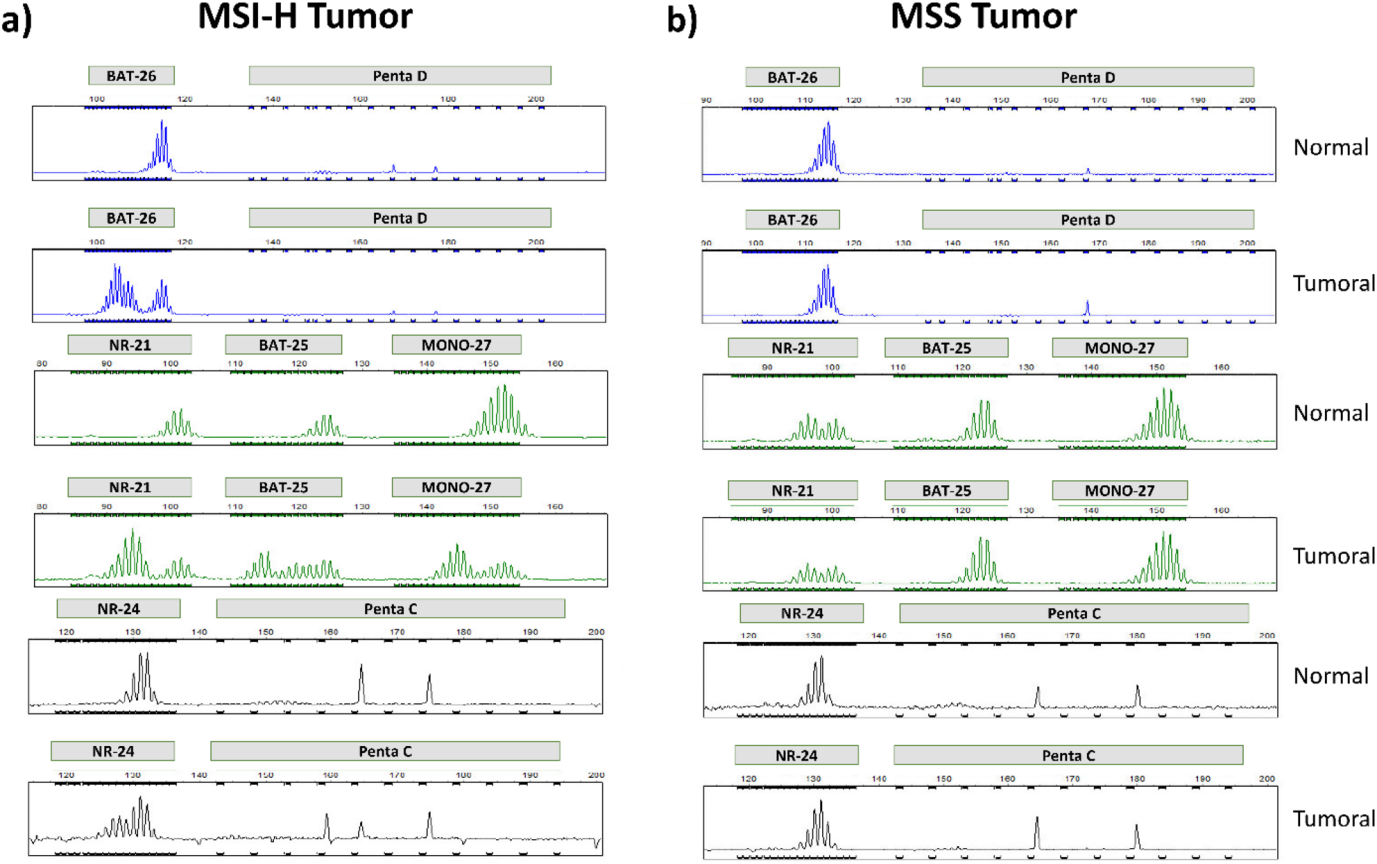
MSI profiles in the MSI-H and MSS patients after multiplex-PCR and fragment analysis achieved for (**a**) GC cancer patient with a high level of microsatellite instability in all 5 mononucleotide repeats, and (**b**) GC patient with stability in microsatellite markers.

**Figure 4:**
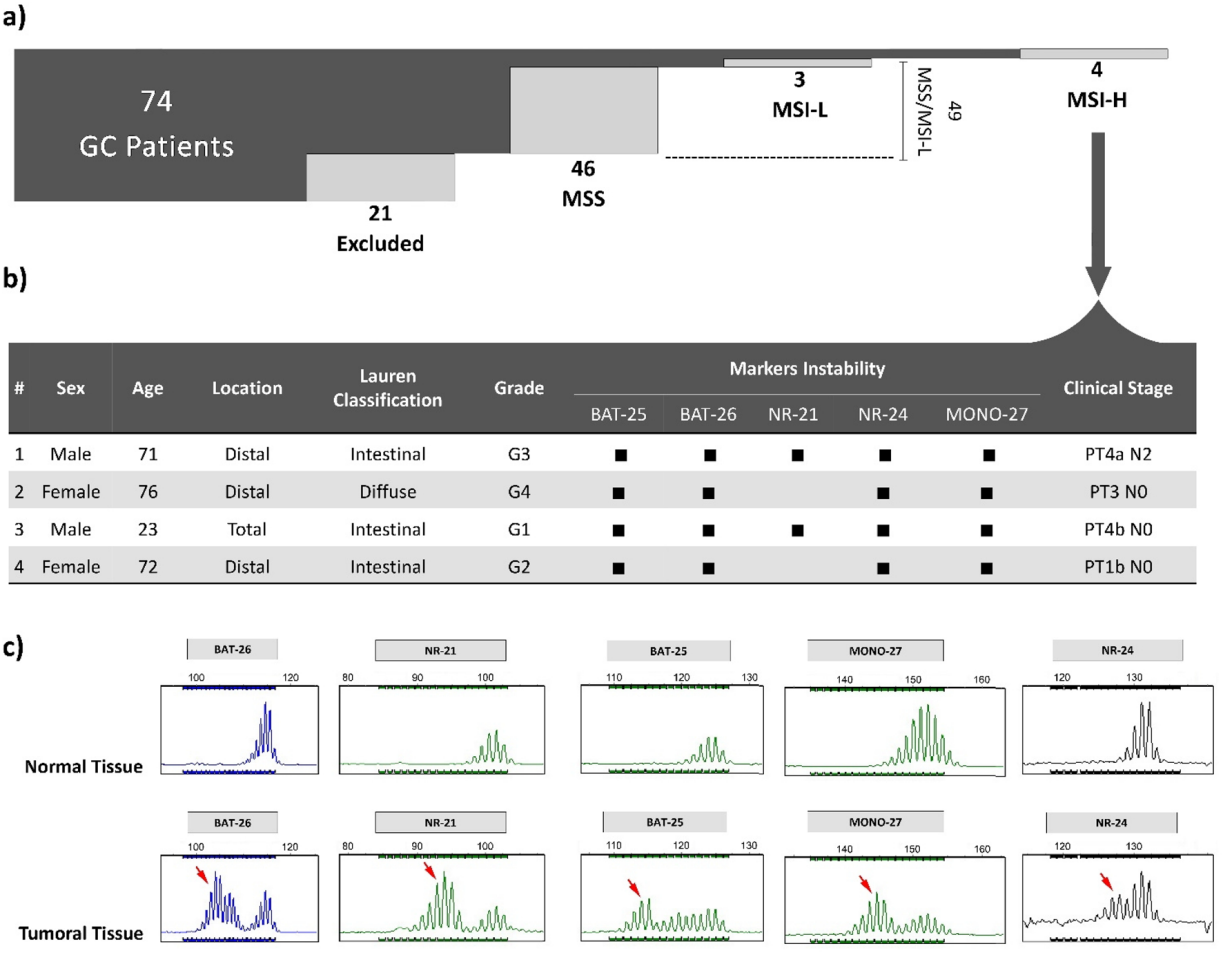
(**a**) Samples categorization based on MSI status and (**b**) clinicopathological characteristics of patients with MSI-H along with the MSI loci PCR products profile of allelic alterations achieved in four tumoral samples. In two samples, instability was observed in all five loci, while in the other two samples, instability was detected in four loci. (**c**) The fragment analysis result represents MSI-H status. The red arrow points to the left shift, indicating the markers with high microsatellite instability (MSI-H).

The electropherograms of MSI-H and MSS patients are demonstrated in Fig. 3. As shown, in the MSI-H sample, the peaks of the microsatellite markers have shifted to the left (Fig. 3a), indicating a deletion in the length of the corresponding region resulting from the DNA polymerase slippage. Such an error during DNA replication could remain in the absence of a functional MMR system. Conversely, in the MSS electropherogram, both tumor and normal microsatellite markers revealed identical patterns (Fig. 3b). Based on the obtained results, 4 out of 53 GC patients (i.e., 7.5% of the samples) showed MSI-H phenotype, while three cases (i.e., 5.7% of the samples) exhibited the MSI-L phenotype. The remaining 46 cases (i.e., 86.8% of the samples) revealed the MSS phenotype. For statistical analysis, the MSI-L was treated as MSS. Therefore, 7.5% of gastric cancer patients possessed MSI-H status and 92.5% were considered MSS (Fig. 4a). Figure 4b provides a detailed overview of the clinicopathological characteristics of the 4 MSI-H samples. Additionally, the schematic of mutations in MSI markers of a 23-year-old patient (#3 in Fig. 4b) has been demonstrated in Fig. 4c, in which arrows have marked the instabilities.

The statistical analysis based on clinicopathological variables and MSI status has been summarized in Table 1. The reported results showed that MSI-H was more likely to occur in older individuals. In the population of study, ignoring the 23-year-old young man, the other MSI-H phenotypes were observed in patients aged 71, 72, and 76 years. Furthermore, statistically, the MSI-H phenotype was more associated with older ages (> = 68) (p‑value = 0.0015) and lower lymph node involvement (p-value = 0.0004). However, although MSI-H was more observed in intestinal type, female sex, and distal location of the tumor, such associations could not be considered significant.

**Table 1:**
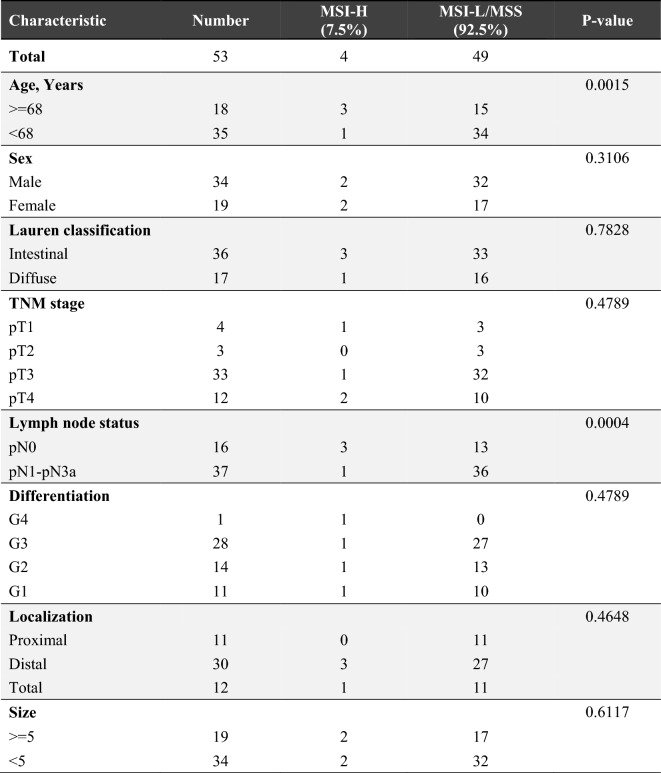
Association between MSI status and clinicopathological characteristics in GC patients.

## Discussion

Gastric cancer is a multifaceted disease characterized by diverse genotypes, phenotypes, and clinical outcomes. Despite advancements in treatment options, desired survival rates have not been achieved. In addition to the challenges in developing effective therapies, there is a lack of diagnostic and predictive biomarkers to guide treatment selection. It should be noted that with respect to the advancements in precision medicine and targeted therapeutic approaches, the opportunity for extended survival following accurate cancer diagnosis would be much higher^20,21^.

According to the informative phenotypic and genotypic variations correlating with clinical data, new therapeutic strategies could be developed in the precision and personalized medicine era. Achieving this goal requires the identification and classification of molecular alterations. By doing so, different biomarkers can stratify prognosis and predict treatment response could potentially be introduced. This would enable selecting the most appropriate treatment method avoiding nonspecific and harmful therapies. MSI, a molecular subgroup of GC, is considered in both TCGA and ACRG classifications^10,11^ and has been recognized as a predictive biomarker for immunotherapy response and is associated with a better prognosis and a lower likelihood of tumor relapse. Given that gastric cancer has the highest incidence in the Asiatic population, detailed information on the prevalence of MSI and its relationship with clinicopathological parameters in retrospective studies would be of utmost importance. However, such information is currently unavailable thus, more research must be conducted to promote knowledge and understanding in this regard^5,22^.

Our study aimed to evaluate the incidence of microsatellite instability in gastric cancers within the Iranian population and explore its association with clinicopathological characteristics. We analyzed MSI status in 53 gastric cancer patients to achieve this. We employed standardized protocols for assessing MSI status and collected detailed clinicopathological data for each patient. However, the sample size may be considered relatively small, it is important to emphasize that our study provided valuable insights into the incidence and clinicopathological features of MSI in the specific Iranian population under investigation.

In this study, by using a five mononucleotide repeats panel, MSI status was analyzed in Iranian GC patients. Based on the obtained results, a frequency of 7.5% (4 out of 53 GC patients) exhibited MSI-H phenotype. Although this incidence is relatively lower than the reported prevalence of MSI-H in GC patients by ACRG and TCGA, it is consistent with those reporting a prevalence of less than 10% (Table 2) in other populations worldwide^4,23–29^. As for the Middle Eastern countries, a prevalence of 11.6% has been reported in Turkey^30^. However, due to the lack of comprehensive MSI evaluation in the Middle East, the exact prevalence could not be determined. Therefore, the detailed information in our study regarding the MSI-H GC incidence in Iran, as a Middle Eastern nation, holds significant importance in contributing to the existing literature on gastric cancer and MSI in this region.

**Table 2:** Reported datasets on the microsatellite instability and association with clinicopathological variables.

#	MSI-H	Samples	Clinicopathological Variables								Refs.
			Older Ages	Female Sex	Intestinal Subtype	TNM Stage	Less Lymph Node Involvement	Differentiation	Distal Location	Bigger Size	
1	7.5%	226	0.074	NA	NA	NA	0.0036	NA	NA	NA	^4^
2	5.6%	414	0.010	NA	0.028	Increased T stage (0.009)	NA	NA	NA	0.014	^23^
3	8.5%	1990	0.001	< 0.001	< 0.001	Lower tumor stage (0.001)	NA	Differentiated histological type (0.001)	0.001	NA	^26^
4	8.1%	310	0.008	-	0.003	NA	–	–	–	–	^27^
5	9.8%	102	NA	NA	NA	Lower clinical stages (I–II)	NA	NA	NA	NA	^28^
6	8.2%	328	< 0.001	NA	0.017	NA	NA	NA	< 0.001	0.011	^29^
7	9.5%	327	0.009	NA	0.016	Lower pTNM stage (0.017)	0.004	NA	0.022	0.022	^44^
8	7.5%	53	0.0015	NA	NA	NA	0.0004	NA	NA	NA	This study

Statistical significance has been represented in terms of p-value.

NA, no association.

Table 3 illustrates the prevalence of MSI in East Asia, encompassing Korea, China, Japan, Taiwan, and Malaysia. Extensive research has been conducted on MSI status, particularly in South Korea. A recent study involving a large cohort of 838 GC patients revealed a prevalence of 11.9% in East Asia^31^. Similarly, in China, a study involving 1757 gastric cancer patients reported a prevalence of 10.5% for MSI-H^32^. In a South Asian study conducted in India, a prevalence of 40% was reported^33^ in which BAT-25, BAT-26, and dinucleotide markers of D2S123, D17S250, D16S752, D16S265, D16S398, D16S496, D18S58, and D16S3057 were utilized to identify MSI-H phenotype. Additionally, MSI-H has identified whether at least two markers were unstable or just instability was observed in the BAT-26 Maker.

**Table 3:**
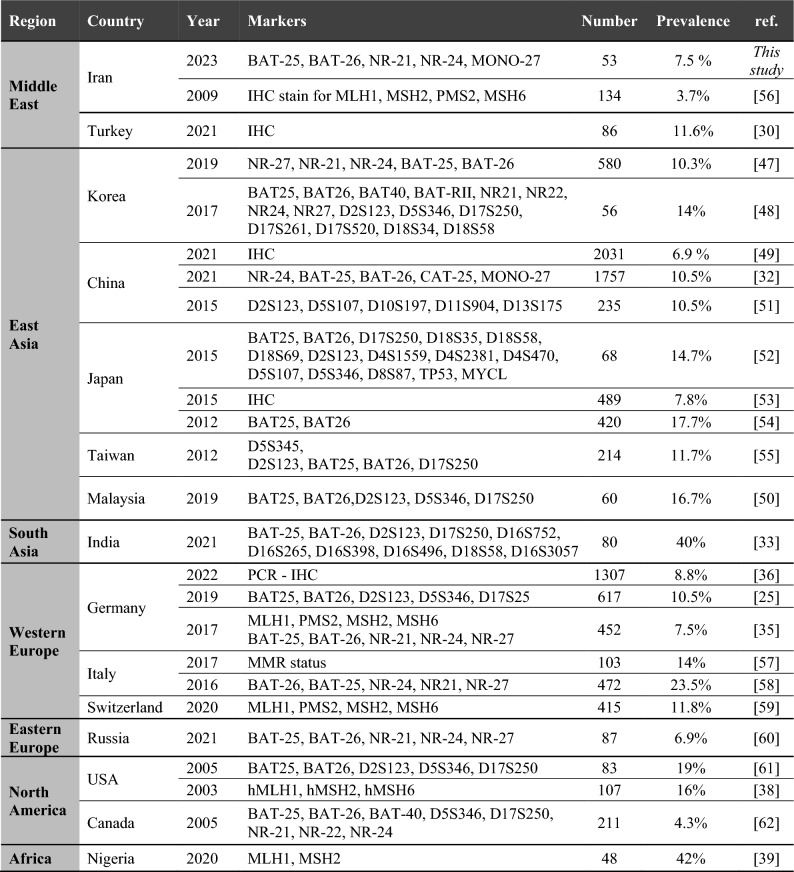
Prevalence of MSI-H phenotype in different regions based on different MSI markers and detection methods.

Therefore, it can be inferred that such high reported prevalence could be attributed to broader criteria used for detecting MSI-H^34^. Prevalence in Western European countries such as Germany, Italy, and Switzerland is summarized in Table 3. In Germany, a similar approach to this study was employed, where a panel of 5 mononucleotide repeats was used to evaluate 452 GC patients, resulting in a prevalence of 7.5% for MSI-H^35^. In 2022, another study conducted in Germany involving a large cohort of 1307 GC patients reported a prevalence of 8.8%^36^. Additionally, a study on German Caucasian patients revealed a prevalence of 7.3% in this particular region^37^.

In North America, a prevalence of 16% has been reported among lymphocyte-rich gastric cancers based on the lack of staining for hMLH1, hMSH2, and hMSH6^38^. In a recent report focusing on Africa, a small sample size of 42 Nigerian patients showed a high frequency of 42% MSI-H determined by the MLH1 and MSH2 immunostaining status^39^. Some previous studies have reported MSI-H prevalence exceeding 30% in the population affected by GC^19,40^. However, some of these studies, utilized microsatellite markers that are less sensitive in detecting MSI status, such as dinucleotide repeats^5,41,42^.

Numerous efforts have been made to identify the association of MSI with different clinicopathological features in order to characterize the MSI subgroup better. Several studies have demonstrated an association between MSI-H GC and specific clinicopathologic variables, including older ages, intestinal type according to the Lauren classification, lower lymph node involvement, distal location of the tumor, lower pTNM stage, and smaller tumor^29,43,44^. These associations with different clinical variables in MSI-H cancers are expected as these tumors are believed to primarily arise and progress through the accumulation of genetic changes rather than any other pathways.

The results of this study align with the existing literature, as they indicate a significant association between MSI-H and older ages (our analysis demonstrated a significant association between ages above 68 and MSI-H (p-value = 0.0015)). Similar conclusions have been drawn in other studies^29^ and a strong association (p-value < 0.001) between MSI-H phenotype and older age (> 70) has been reported. In the study conducted by Oki et al.^45^, the mean age of MSI-H patients was 67.8, while in MSS patients the mean age was 63.3. Kim et al.^23^ also presented a mean age of 64 for MSI-H patients compared to 58.59 for MSS patients, further confirming the association (p-value < 0.05) with older ages. This association has also been observed in Russia, specifically in terms of deficiencies in the MMR system. In that context, the mean age of dMMR patients was found to be 69 (p-value = 0.008)^27^.

To better understand MSI-H status in GC, the association between MSI-H status and lymph node involvement was evaluated in this study. The finding concluded that MSI-H GCs were significantly associated with lower incidence of lymph node involvement (p-value = 0.0004). Accordingly, it could be inferred that the absence or reduced lymph node involvement may be indicative of a favorable prognosis in patients with MSI-H tumors. This aligns with similar results presented in previous studies. For instance, Huo et al.^4^ reported a significant association between MSI-H and the absence of lymph node involvement (p-value = 0.0036). Additionally, Lee et al.^44^ demonstrated a significant association between MSI-H and a lower prevalence of lymph node metastasis (p-value = 0.004). Furthermore, a comprehensive meta-analysis published in the literature^46^ confirmed the consistent observation of less lymph node involvement in MSI-H tumors and a significant association between MSI and the absence of lymph node metastases (p-value < 0.001). These collective findings support the notion that the presence of MSI-H status in gastric cancer may suggest a more favorable lymph node profile and potentially a better clinical outcome.

In contrast, the literature contains conflicting reports on the relationship between MSI status and the TNM stage. The controversial disassociation with non-metastasis GC observed for the samples taken after surgery could be justified by the fact that the hematogenously metastasized gastric carcinomas do not commonly undergo gastrectomy. Consequently, the available data on the association between MSI-H and TNM stage make it challenging to draw a reliable conclusion. In addition, considering that MSI-H status is associated with a good prognosis, it is not highly expected to find patients with TNM stage 4 and MSI-H status^24^. In light of the findings from this study, which included two MSI-H patients among 12 TNM stage 4 samples, it is suggested to analyze metastatic GC patients for the MSI-H status identification.

The study's results indicated that, while some studies have reported associations, not all clinical and pathological factors are significantly associated with MS-H, and such relationships remain ambiguous. Significant associations could not be inferred for factors such as gender, Lauren classification, differentiation, localization, and tumor size. However, based on the presented data, MSI-H was more frequently observed in the females, cases classified as intestinal type according to Lauren classification, tumors located in the distal region, and bigger tumor size.

One issue restricting the applicability of the detection of MSI in GC patients is the diversity of microsatellite markers and the variety of commercial panels proposed for MSI status assessment^4^. These panels include Bethesda panel, consists of two mononucleotide markers (BAT-25 and BAT-26) and three dinucleotide markers (D2S123, D5S346, and D17S250), Promega MSI analysis system of five nearly monomorphic mononucleotide repeat markers (BAT-25, BAT-26, MONO-27, NR-21, and NR-24), Idyll MSI test with 7 MSI loci (ACVR2A, BTBD7, DIDO1, MRE11, RYR3, SEC31A, and SULF2), the Titano MSI system including Bethesda panel BAT25, BAT26, D2S123, D17S250, D5S346, and BAT40, D18S58, NR21, NR24, TGFβRII, as well as wide ranges of novel MSI markers introduced in other studies^63,64^. As a result, there is no consensus on the specific panel of markers to determine MSI in GC, and the assessment of MSI status has been evaluated using different sets of microsatellite markers. Further, the final determination could be influenced by the number of markers utilized to detect MSI, particularly when it comes to differentiating MSI-H and MSI-L based on the percentage of unstable markers. This concern could also be addressed by employing larger multiplex PCR panels or even NGS techniques. These approaches would enhance sensitivity and accuracy by evaluating more microsatellite markers, thus reducing the impact of false positives and false negatives on final determination.

In GC, as the MMR and microsatellite genes are susceptible to a wide range of initial mutation ^63,65^, highlighting the significance of investigating MSI markers and considering ethnic-based differences. This suggests further efforts to identify more specific MSI markers for detecting MSI-H GC. To the best of our knowledge, observed variations in prevalence across different studies, alongside using different MSI detection panels and methods, might be attributed to the intra- and inter-population genetic variabilities.

Although the number of samples was limited, the results confirmed the significant correlation between MSI status and certain clinicopathological variables, such as older age and fewer lymph node involvements. However, it should be noted that the small sample size could influence the other variables of no statistical association. Therefore, obtaining more samples could enhance the reported results, leading to a more accurate and comprehensive conclusion regarding the necessity of implementing the MSI status detection test for all gastric cancer patients. Additionally, to further investigate the role of MSI in gastric cancer, it is essential to conduct follow-up studies on patients with and without treatments.

## Conclusion

The microsatellite instability status of a group of Iranian gastric cancer patients was assessed in this study due to the limited information available on microsatellite instability in gastric cancer in the Middle East. The findings revealed the microsatellite instability occurrence rate of 7.5% which was strongly associated with specific clinicopathologic characteristics like older ages and fewer lymph node involvements. The low incidence of MSI-H and its correlation with clinicopathological features in the gastric cancer patients of this study should be interpreted in conjunction with the results of the other studies that have reported similar low MSI-H prevalence to provide realistic predictions for the global MSI ratio in gastric cancer.

## Data Availability

Data reported in this manuscript are available on request by contacting the corresponding author.
